# Competitive Advantage in Intercollegiate Athletics: Role of Intangible Resources

**DOI:** 10.1371/journal.pone.0145782

**Published:** 2016-01-05

**Authors:** Doyeon Won, Packianathan Chelladurai

**Affiliations:** 1 Yonsei University, Seoul, Korea; 2 Troy University, Troy, Alabama, United States of America; University of Rome, ITALY

## Abstract

The present research explored the dynamics of competitive advantages in intercollegiate athletics by investigating the contribution of intangible resources (i.e., athletic and academic reputations) on the generation of more tangible resources (i.e., human and financial resources), which in turn influence the athletic performance (i.e., winning record) and academic performance (i.e., graduation rates), and gender equity. The research was based entirely on archival data of 324 NCAA Division I member institutions. The results of the SEM supported the study’s basic arguments that tangible resources are the sources of competitive advantages in Division I intercollegiate athletics, and that intangible resources contribute to the generation of tangible resources.

## Introduction

The importance and impact of intercollegiate athletics governed by the National Collegiate Athletic Association (NCAA) in America are enormous in every aspect of American society. Apart from the fact that millions of people attend the NCAA contests [1], and that millions of dollars are involved at the individual institutional level [2], intercollegiate athletics has a profound effect on the universities that participate in intercollegiate athletics. The social impact of intercollegiate athletics is seen in the cultivation and maintenance of university tradition and community relations [3,4].

The public image and the prestige of universities are altered by the successes and failures of their athletic programs [5,6]. Media exposure of athletic teams has not only generated revenue for institutions but also has made institutions more attractive to potential students [7,8]. Thus, athletic success has implications for institutional outcomes in the context of higher education, such as larger pool of prospective students, and bigger endowments for academic or athletic purposes [9]. In sum athletic programs often play an important role in public relations and promotion of the university community as a whole including better student recruitment, alumni relations, and school life, and more alumni donations and endorsements [7,10]. It is not surprising, therefore, that universities have increased their efforts to develop the athletic departments with a view to creating winning teams in different sports.

Despite these efforts, only a few of the 324 Division I institutions in the NCAA and 115 Division I-A (currently known as Division I-FBS, football bowl subdivision) member institutions are thriving while others are struggling in terms of athletic, financial or educational success [11,12]. For example, most of NCAA Division I-A, I-AA, and I-AAA institutions were in financial deficit of $600,000, $3,400,000, and $2,800,000 respectively. Without institutional support, only 35% of Division I-A, 8% of Division I-AA, and 7% of Division I-AAA members created surpluses [11]. Only 23 institutions in the Division I-FBS (I-A) reported positive net surplus in 2012 [12].

Aside from financial successes, there are other performance differences among the universities. In terms of the Learfield Sports Directors’ Cup rankings (formerly, Sears Directors’ Cup), several Division I members such as Stanford, UCLA, and Michigan have produced winning programs over competing institutions [13]. In terms of student-athletes’ graduation rate, such NCAA members as Syracuse (81%), Stanford (86%), and Virginia (84%) have been successful while other member institutions such as Oklahoma State (24%), Florida (41%), and Louisville (35%) show poor graduation rates of student-athletes [14]. In terms of newly implemented graduation success rate (GSR) and academic progress rate (APR), some universities like Central Florida, Miami, and Wisconsin have been successful while other universities or athletic programs received the NCAA penalties due to the unsuccessful performance in APR [15].

These differences in the effectiveness among institutions of similar sizes and types beg the question of why some athletic departments are successful in achieving some of their goals while others fail to do so. In other words, why are some departments achieving competitive advantages over others in different spheres of endeavor? One explanation for the successes of some departments and failures of others can be gleaned from the recently advanced strategic approach of resource-based view of organizations.

### Resource Based View (RBV)

The main focus of the field of strategic management is on “how firms achieve and sustain competitive advantage” (p. 509) [16]. In regard to the search for the source of sustainable competitive advantage, the resource-based view has gained prominence in the last three decades. The central tenet of the RBV is that sustainable competitive advantage of the organization comes from resources under its control and how those resources are used [17]. Thus, this theory encourages organizations to obtain critical resources, utilize those resources productively, and develop proactive strategies based on the analysis of resources [16,17]

In RBV, an organization is seen as a bundle of productive resources [18]. The RBV theory starts with the assumptions that (a) organizations in a given industry may have heterogeneous resources and (b) the resources are imperfectly mobile [17]. The two characteristics of *resource heterogeneity* and *imperfect mobility* make some of those resources *valuable*, *rare*, *imperfectly imitable*, *and hardly substitutable* [17]. The extent to which a resource is valuable, rare, imitable, and substitutable is indicative of its potential to generate sustainable competitive advantage over other organizations in the same field [19,20].

### *RBV in Intercollegiate Athletics* (ICA)

Smart and Wolfe [21] used Barney’s [17] resource categorization to investigate the plausible resources as sources of competitive advantage in the Big Ten football programs. An athletic program’s success was defined as a package of (a) winning (e.g., win-loss records), (b) educational (e.g., student-athlete graduation rate), (c) ethical (e.g., absence of NCAA rule violations), and (d) financial (e.g., financial surplus) aspects of each program. Smart and Wolfe [21] argued that the Penn State football program had sustainable competitive advantage over competitors in the field as it was considered to be the most successful team among Big Ten football programs in the 1990s. Further, they argued that organizational resources such as the Penn State football team’s history and culture (operationalized as the coaching staff’s long tenure) were the sources of sustainable competitive advantage. In their view, physical resources (e.g., stadium, training facilities and equipment, dormitories, information management systems for recruiting and game analysis) and human resources (e.g., athletic ability of student-athletes, coaches’ experience) are valuable yet not rare in comparison to competitors. In a later study, Smart and Wolfe [22] investigated the effects of leadership and human (i.e., player) resources on performance in Major League Baseball. They included manager’s age, prior MLB managerial experience, previous managerial winning percentage, number of MLB teams previously managed, number of years managing the current team, previous Manager of the Year awards, and In-Year managerial change as indicators of leadership while performance was indicated by winning percentage. They found that player resources accounted for 67% of the variance in performance while leadership contributed less than 2% of the variance.

Even more recently, Wolfe, Wright, and Smart [23] have employed the RBV to discuss how Oakland Athletics, a franchise in Major League Baseball, was able to gain a competitive advantage by employing an innovative player evaluation technique called *sabermatics*. Sabermatics is “a statistically based approach for developing and applying objective knowledge to baseball….[it] is an important determinant of player evaluation and of “in-game” tactics (p. 113). Wolfe et al. [23] suggest that Oakland Athletics enjoyed a competitive advantage because of two unique resources—the knowledge generated by the use of sabermatics and, thereby, the recruitment of quality players at low cost.

Cunningham [24] investigated the impact of human resources on the performance of Division I athletic departments. He used the average of the coaches’ salaries and the recruiting budget as operational measures of human resources and the Sears Directors’ Cup scores as a measure of performance. In another study, Cunningham and Sagas [25] found that human resources (operationalized as coaching experience and racial diversity of coaching staff) was a significant predictor of NCAA football program success. Berman, Down, and Hill [26] investigated tacit knowledge (defined as shared experience among players) and team performance of NBA teams. They found a curvilinear relationship between the two entities. That is, as shared experience increased team performance increased up to a certain point. When shared experience went beyond that point team performance decreased. In a case study, Anderson and Birrer [27] claimed that the primary resource that has contributed the sustainable success of the Gonzaga University’s basketball program is ‘Sweet Sixteen’ resource, along with some strategic decisions made by the administration.

While these studies represent pioneering efforts in employing the resource-based view in investigating competitive advantage in professional sports and collegiate athletics, they are rather narrow in their scope. Either the studies focus on a single athletic program [21,23] or they studied the influence of one or two resources [24–26]. As Carmeli and Tishler [28] noted, while studies with a single [or two] resource are valuable, they have some limitation in that “competitive position is derived from a combination of several resources and capabilities” (p. 301). In line with this stance, the present study investigated the influence of several types of resources in competitive advantage of several universities and across different goals.

### Classification of Resources

A useful first step in the study of resources as sources of competitive advantage is to categorize the resources and assess the status of an organization in terms of those categories [16, 29–32]. Barney [17,33] categorized firm resources into *physical capital* resources (e.g., physical technology, plant and equipment, geographic location), *human capital* resources (e.g., experience and knowledge of individuals associated with a firm such as sales personnel), *financial capital* resources (e.g., debt, equity, and retained earnings), and *organizational capital* resources (e.g., history, relationships, trust, and organizational culture).

While Barney’s classification has been well received, there is also the suggestion that resources be classified on the basis of their *tangibility* [34–36]. Examples of intangible resources include trademarks, patents, copyright, registered designs, contracts, trade secrets, reputation, and networks as well as know-how. Tangible resources include all physical items that the organization possesses, such as facilities, raw materials and other equipment [34,35]. The distinction between tangible and intangible resources underscores the notion that intangible resources are more valuable than the tangible resources in most cases. As Hall [35] illustrated, the price of a firm’s stock is the reflection of the firm’s tangible (e.g., finances, facilities) and intangible resources (e.g., reputation). However, intangible resources of a firm play a bigger role in determining a market price of a stock in comparison to tangible resources.

Based on empirical studies, Hall [35] argued “intangible resources are the ‘feedstock’ of the four capability differentials” (p. 143). The four capability differentials refer to (a) functional (e.g., knowledge), (b) positional (e.g., reputation), (c) cultural (e.g., organizational culture), and (d) regulatory differentials (e.g., contracts). Among these, the intangible resource of reputation is critical in achieving competitive advantage because highly reputed organizations can differentiate their products or themselves via their reputation [35]. As intangible resources are unobservable, they represent higher barriers to imitation [37], and hence, “intangible, more than tangible, resources have potential for competitive advantage creation” (p.112)[34]. To illustrate, intercollegiate athletic departments register their logos, which are then licensed to other commercial firms. Hall [35] would view the registered right to the logo itself as an intangible asset. To the extent the demand for the use of the logo and the sale of licensed goods are based on the reputation of the university, the reputation becomes even more of an intangible asset. Thus, a university’s reputation cannot be duplicated by another while the process of creating a logo and licensing it to generate revenue can be easily copied.

The foregoing analysis of intangible resources leads to the suggestion that the intangible resources of the organization may indeed be the *contributing* resources in the sense they facilitate the garnering of the more tangible resources. For instance, the good will toward the university (an intangible and contributing resource) would be reflected in the endowments (tangible resource) bestowed by well-wishers. In fact, studies in alumni donations have suggested that alumni contributions are, to a great extent, the function of the institutional characteristics [38]. Thus, consistent with Hall’s [35] perspective, the current study conceives of the intangible resources as contributing resources that facilitate the generation of the more tangible resources. As most of intangible resources fall under Barney’s [17] category of organizational resources, the current study will use the label “organizational resources” and “intangible resources” interchangeably.

### Resources in Intercollegiate Athletics

We have followed Barney’s [17] original categorization in specifying the following resources for investigative purposes. They were grouped into organizational, physical, and human resources. As Barney’s [33] fourth category of financial capital resources relating to debt, equity, and retained earnings is more germane to profit oriented business enterprises, we have not included it as a separate category. Instead, we have combined the finances available to a university athletic department with its physical resources and labeled it the financial resources as was indicated by Putler and Wolfe [39]. The following section describes the types of resources selected for the study.

#### Organizational Resources (Intangible Resources)

Athletic reputation. Reputation is defined as “the evaluation of a firm by its stakeholders in terms of their affect, esteem, and knowledge” (p. 1093)[40]. This reputation in the context of universities may relate to their athletic as well as their academic endeavors. Athletic tradition is built on the excellence exhibited by the athletic teams over a number of years as reflected in the number of championships won over the years. Championships allow an athletic department to promote itself and the university to critical stakeholders and thus cultivate the reputation. Therefore, the cumulative number of NCAA championships of each athletic department would be a good indicator of athletic reputation. It is noted that because the unit of analysis of the current study is each athletic department rather than a specific athletic team, the cumulative championships of all sports is the measure of interest.

Academic reputation. This variable concerns academic excellence of institutions in which athletic departments are housed. Academic reputation and standing has been the basis for classification of universities (e.g., Peterson’s College Guide). It is not uncommon to gauge the academic reputation of universities by the difficulty of gaining entrance to them. For instance, the Peterson’s College Guide provides entrance difficulty data for all institutions associated with NCAA Division I.

Tradition (History). The position and behavior of the organization is often decided by the past experience of the organization [16,17]. Likewise, an organization’s current levels of resources and capabilities can be a function of the paths (i.e., decisions and directions) it had taken in the past in reaction to the then existing environmental contingencies [41]. Such resources and capabilities tend to be inimitable and nontradable in so far as they represent unique organizational routines [17,42] as well as unique organizational culture [43]. Accordingly, the history of the institution is one of the organizational resources.

#### Financial Resources

Financial and physical resources are fundamental to any organization in any context. In the context of ICA, these resources are required to run the athletic program in general, operate individual athletic teams, organize athletic events, recruit athletes, and offer scholarships to them. Some of the resources such as financial resources are more transferable. Financial resources are easily transferable to physical resources. We used the following as the indicators of financial resources.

Sports Expenses. According to the U.S. Department of Education, sport expenses are defined as all expenses attributable to athletic activities, including athletically related student aid, recruiting expenses, salaries and benefits for coaching staff, and team expenses.

Non-Sports Expenses. The expenses not allocated by sport refer to expenses such as administrative costs, appearance guarantees and options, contract services, fundraising activities, promotional activities and salaries for administrative staff members (excluding coaches’ salaries).

#### Human Resources

Number of coaches. The term is defined as the total number of coaching staffs associated with each athletic department. While the greater numbers of employees do not always indicate a better quality of human resources at an organization, universities tend to hire coaches with expertise, experience and diverse perspectives on coaching.

Number of athletes. The term is defined as the total number of student-athletes in each athletic department. Although the rules of a particular sport specify the number of athletes on the roaster for an event, universities are at liberty to carry more or less number of athletes. The larger the number of athletes, the greater is the possibility of the emergence of high performers. This is particularly true in collegiate ranks as the athletes are still in their growth and development stage. It is not uncommon to see substitutes and benchwarmers from one year becoming superstars in the next year.

### Goals in Intercollegiate Athletics

The emphasis on resources is based on the notion that these resources are required to attaining organizational goals. Accordingly, the relevance or criticalness of a given resource varies across prioritized organizational goals or mission of a given organization. For instance, the critical resources of a manufacturing firm (e.g., a manufacturer of furniture) would be different from those of a service firm (e.g., a restaurant). In our context, some of the critical resources required by an intercollegiate athletic department would differ from those required by, for example, a city recreation department. A profit-oriented professional sport franchise would require certain critical resources that may be different from those required by an intercollegiate athletic department.

An extension of the above perspective would suggest that even within a single organization (or a group of organizations), different resources would become critical to achieve different goals it may pursue. In our context, ICA departments have two sets of goals, namely educational and athletic goals [44–46]. Trail and Chelladurai [46] grouped 10 goals of ICA into (a) performance goals (i.e., winning, entertainment, visibility and prestige, financial security, and national sport development) and (b) developmental goals (i.e., academic achievement, health/fitness, social/moral citizenship, careers, and culture of diversity).

For the purposes of the present study, we identified three critical goals for intercollegiate athletics—overall athletic success of the program, the overall academic success of the program, and the success in fostering gender equity within the program. While the former two goals (i.e., athletic and academic goals) have garnered most attention, the goal of gender equity is equally important. The emphasis on gender equity as a goal for NCAA athletic departments was greatly increased with the promulgation of Title IX. Subsequently, university administrators and the NCAA have touted the virtue of gender equity and have strived continuously to foster it. Given the trimodal foci of ICA goals, the study was concerned with identifying the resources that were more associated with attainment of each of the three goals.

### Model of Resources and Outcomes in Intercollegiate Athletics

**Fig 1:**
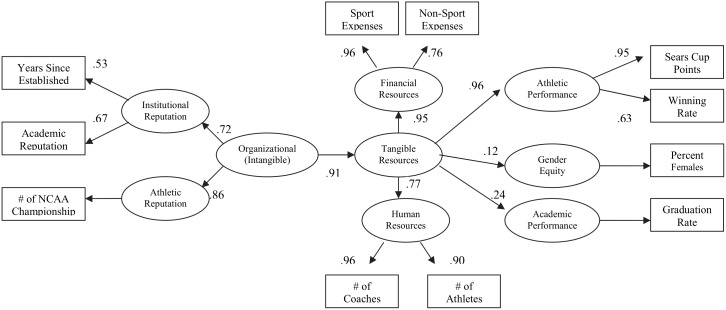
A Proposed Model of Resources in ICA.

The proposed model for empirical verification is shown in Fig 1. The model incorporates three broad categories of resources (*organizational*, *financial*, and *human resources*), and three outcomes (*athletic performance*, *academic performance*, *and gender equity*). The model suggests that organizational resources are the driving force behind the generation of the other two forms of resources—financial and human resources. The latter two forms of resources are said to influence both the performance and developmental outcomes.

#### Alternative Model

**Fig 2:**
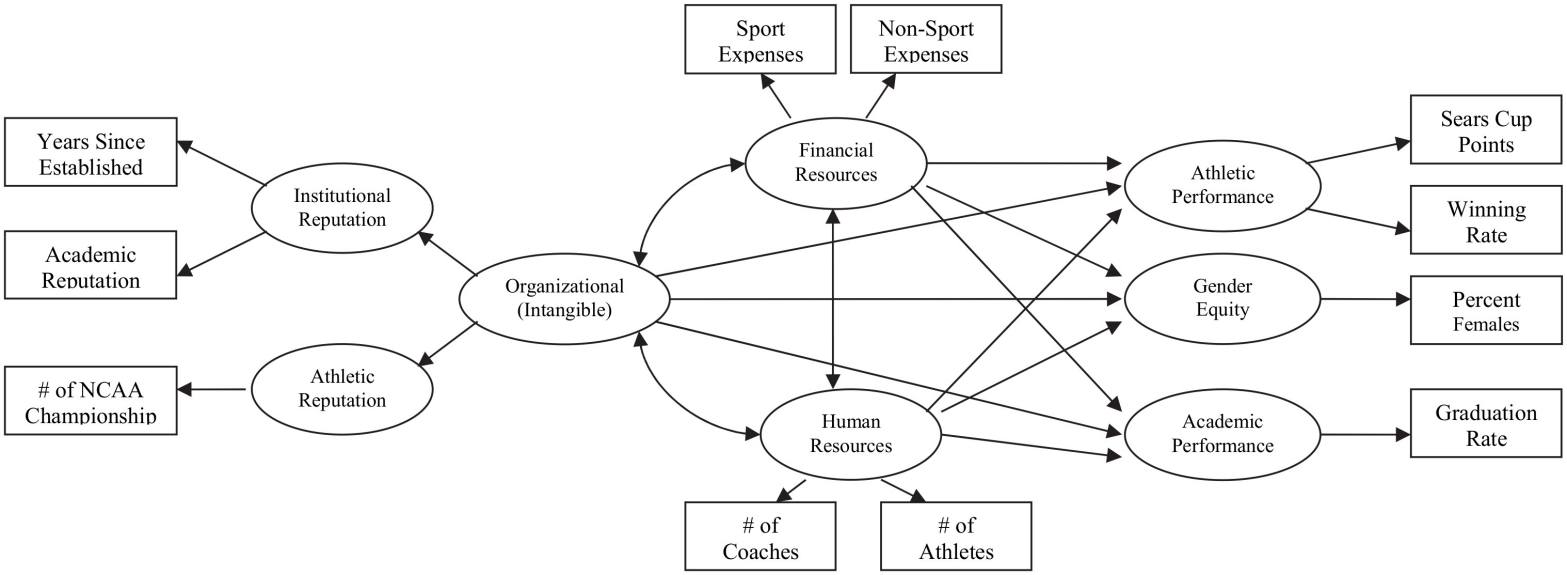
An Alternative Model of Resources in ICA.

The alternative model included in the present study takes a different conceptual approach in which intangible resources (i.e., organizational resources) are placed parallel with tangible resources (i.e., human and financial resources). That is, organizational resources are not considered as contributing resources. Rather, the alternative model sees both tangible and intangible resources independently influencing the attainment of intercollegiate athletic goals (see Fig 2).

From a different angle, it is also plausible that tangible resources are building blocks of intangible resources given that some of the tangible resources are transferrable while organizations can deploy more strategic moves with affluent tangible resources. However, in the context of intercollegiate athletics, it is more plausible and reasonable to suggest that intangible resources are much more critical in achieving sustainable competitive advantages and success over time [39] because these intangible resources can be considered as the type of assets that contribute in acquiring tangible resources and creating values to the organizations, including athletic departments [34–36].

## Methods

### Ethics Statement

This study was approved by the Institutional Review Board of the Office of Responsible Research Practice at The Ohio State University through the expedited review procedure because this study falls under the exempt research category

### Population and Sample

The 326 universities and colleges that are associated with the NCAA at the Division I level constituted the study population. In that the data on the study variables for all schools in the NCAA Division I were available, the target and accessible populations were identical [47]. However, two institutions were excluded from the study because there was no record available for these two institutions. Thus, the size of the final pool was 324 institutions comprised of 121 Division I-A (37.3%), 118 Division I-AA (36.4%), and 85 Division I-AAA institutions (26.2%).

### Sources of Data

The necessary data of 324 institutions for the current study were obtained from several archival records to collect the relevant data for the 2003–2004 season. The data regarding athletic operations were drawn from Equity in Athletics surveys for 2003 [2] as well as from the NCAA on-line database and published sources. The data regarding institution-related information were obtained from such on-line databases as *Peterson’s Guide to Colleges and Universities* [48] and *Sperling’s Best Places* [49]. The number of NCAA championships was obtained from the NCAA website [50]. Thus, all the data to be used in the study were ‘secondary (available) data.’ Collecting data from NCAA and NCAA related web sites is consistent with Berman, Down, & Hill [26] who employed the statistics provided by the NBA and Smart and Wolfe [22] who relied on the data of MLB.

### Variables

#### Organizational (Intangible) Resources

As shown in Fig 1, Organizational Resources are indicated by Institutional Reputation and Athletic Reputation. Institutional Reputation was indicated by (a) the number of years since establishment of the university, and (b) Academic Reputation measured as the degree of difficulty of freshman entrance. The cumulative number of NCAA championships was the measure for Athletic Reputation.

#### Financial Resources

An organization’s physical resources consist of, but not limited to, plants, equipment, facilities, and buildings [17, 51] whereas financial resources refer to monetary resources such as retained earnings [17]. In this study, this resource was operationalized by (a) Sport Expenses, and (b) Non-Sport (Administrative) Expenses. The information was obtained from the U.S. Department of Education website [2].

#### Human Resources

Human resources in athletic departments were operationalized by (a) Number of Coaches, and (b) Number of Athletes. The information was collected from the U.S. Department of Education website [2].

#### Intercollegiate Athletic Goals

Achievement of athletic goals was operationalized by Sears Cup scores and Winning Rate. The Sears Cup scoring is a well-accepted measure of cumulative successes of an athletic program which takes into account the relative successes of all the teams within the athletic program. In addition, winning rates of each athletic department (i.e., a school’s winning percentage for all men’s and women’s school to school contests) were utilized as an indicator since a ‘winning program’ is often used as a criterion of athletic success.

The success in achieving the academic goals was operationalized as Graduation Rate. Gender equity was measured by the percent of females in an athletic program. The information about graduation rate, gender equity, Sears Cup scores, and winning rates was collected from the NCAA website [50], the U.S. Department of Education website [2], the National Association of Collegiate Directors of Athletics website [13], and the U.S. News website [14].

### Data Preparation

#### Normality of the Data

In terms of normality of the data, there were instances of true outliers. For example, four athletic departments earned more than 1,000 Sears Directors’ Cup (now known as Learfield Directors’ Cup) points in 2003 while 60 out of 324 athletic departments did not earn a single point as well as the mean score was about 176 points. In so far as such outliers in the data were accurate observations [52], they were included in analyses. We utilized a square root transformation technique to normalize the data [53]. After the data transformation, the absolute value of skewness were smaller than 2.04 and those of kurtosis were smaller than 4.706. These values indicated that non-normality of the data was not an issue [53]. In addition, all the data were standardized for comparability in the interpretation phase as well as for preventing possible inflations of error variances in structural equation modeling.

#### Multicollinearity

In order to assess the extent of multicollineairity in the data, we computed the correlations among the summated means of each latent variable and single-item indicators. These correlations ranging from .50 to .79 are lower than the critical value of .85 indicative of multicollinearity problems [53].

#### Analyses

First, descriptive statistics were calculated to present the basic characteristics of data as well as to prepare data for structural equation modeling (SEM). Both the hypothesized and alternate models were tested through SEM techniques.

## Results

### Descriptive Statistics

Table 1 presents the means and standard deviations of, and the correlations among the variables of the study. Of the 324 institutions, the majority were public institutions (*n* = 218; 67.3%). On average, each athletic department has 254 male and 203 female student-athletes. Each athletic department reported $9.31 million and $4.44 million as the mean sport expenses and non-sport expenses, respectively (median expenses of $4.82 and $2.23 million, respectively).

**Table 1:** Means and Standard Deviations and Bivariate Correlations for the Variables of the Study. Note: Units for Sport Expenses and Non-Sport Expenses are in millions

	1	2	3	4	5	6	7	8	9	10
**1. Athletic Championship**	--									
**2. Academic Reputation**	.34**	--								
**3. Yrs since Established**	.22**	.39**	--							
**4. Sports Expenses+**	.57**	.37**	.33**	--						
**5. Non-Sport Expenses+**	.40**	.21**	.31**	.73**	--					
**6. # of Coaches**	.47**	.52**	.45**	.69**	.56**	--				
**7. # of Students-Athletes**	.45**	.49**	.48**	.64**	.52**	.86**	--			
**8. Sears Cup Score**	.61**	.41**	.31**	.83**	.66**	.66**	.59**	--		
**9. % of Female Participants**	.07	.13*	-.02	.10	.07	.06	.04	.12*	--	
**10. Graduation Rate (%)**	.10	.49**	.35**	.24**	.09	.34**	.32**	.18**	.18**	--
**Mean**	4.37	3.08	120.10	9.31	4.44	51.34	457.39	176.71	58.72	44.17
**Standard Deviation**	11.41	0.76	49.66	7.64	5.66	18.52	199.09	238.67	13.12	7.15
**Minimum**	0	1	32	0.53	0.00	17	138	0.0	27	9.36
**Maximum**	91	5	367	40.10	29.37	124	1,454	1,420.5	91	66.59

*p< .01;

**p < .001

In terms of correlations, Athletic Performance was significantly and positively correlated with all other variables in the study while Gender Equity was significantly and positively correlated only with Academic Reputation and Athletic Reputation. Academic Performance was significantly correlated with almost all variables except Athletic Reputation and Non-Sport Expenses.

### Structural Model

The results of SEM provided good support for the model (*χ*^*2*^*/df* (101.77/ 37) = 2.75; RMSEA = .074, 90% C: .057, .091; GFI = .95; NFI = .95; IFI = .97; and CFI = .97. These values cumulatively indicated that the model fit the data well.

### Model Comparison

As noted, the alternative model specified direct effects of the three types of resources on the three dependent variables (see Fig 2). The χ^2^ difference between two models of 79.87 with 1 degree of freedom was significant (*p* < .001). The fit indices for the alternative model were χ^2^/df = 5.05; RMSEA = .11; GFI = .91, NFI = .91, and CFI = .93. As these values indicate a poor fit, it was discarded, and the hypothesized model was accepted as tenable.

### Path Analysis

Three sets of path coefficients (shown in Table 2) were calculated by maximum likelihood estimation (MLE) to assess the direct and indirect effects of the three types of resources (i.e., financial, human, and intangible resources).

**Table 2:** Decomposition of Standardized Effects for the Model of Resources.

		Endogenous Variable		
Causal Variable	Tangible	Athletic Performance	Academic Performance	Gender Equity
Intangible Resources				
Direct Effect	.91**	--	--	--
Indirect via Tangible	--	.87**	.22*	.11*
Tangible Resources				
Direct Effect	--	.96**	.24*	.12*

*p < .01;

**p < .001.

#### Direct Effects

Intangible resources were strongly predictive of greater tangible resources (*β* = .91, *p* < .001). Tangible resources directly influenced Academic Performance of student-athletes (*β* = .24, *p* < .001) as well as Gender Equity (*β* = .12, *p* < .05). Tangible Resources had the greatest positive effect on Athletic Performance (*β* = .96, *p* < .001).

#### Indirect Effects

Tangible Resources mediated the relationship between Intangible Resources and Athletic Performance (*β* = .87, *p* < .001). That is, Intangible Resources had a positive indirect influence on Athletic Performance. Intangible resources also indirectly increased Academic Performance of student-athletes (*β* = .22, *p* < .001) as well as Gender Equity of female student-athletes (*β* = .11, *p* < .05).

## Discussion

A significant contribution of this study was to conceptualize and demonstrate that organizational resources (i.e., the intangible resources) contributed to the tangible resources in the form of financial and human resources. Our results showed that intangible resources consisting of academic and athletic reputation explained more than 83% of the generation of tangible resources in the collegiate sports context. As Deephouse [40] noted, such reputation is reflected in significant stakeholders’ knowledge of, and liking and esteem for the university. In addition, the reputation also is “the product of years of demonstrated superior competence” (p. 616)[36].

We also found that the intangible resources were more predictive of an athletic department’s financial resources (*β* = .86, *p* < .001) than its human resources (*β* = .70, *p* < .001). In this regard, it must be noted that the NCAA rules and regulations place some constraints on the number of personnel (i.e., coaches and athletes) for a given team. From this perspective, athletic departments are more restricted from having larger pools of human resources while such restrictions are not imposed on financial resources. A counterpoint to the above reasoning is that the NCAA does not place any restriction on the number of teams an athletic department may support. That is, athletic departments with higher levels of intangible resources could support a relatively larger number of teams than the departments with less intangible resources.

As hypothesized, tangible resources were significantly predictive of the amount of athletic performance (*R*^*2*^ = .91). As noted, intangible resources had an indirect influence on athletic performance (*R*^*2*^ = .75). However, the effects of tangible and intangible resources on academic performance were not as large (*R*^*2*^ = .06 and *R*^*2*^ = .05, respectively). The effects of tangible and intangible resources on gender equity were the weakest (*R*^*2*^ = .01 and *R*^*2*^ = .01, respectively).

It is of concern that the predictive power of resources on attaining academic goals was smaller in comparison to athletic performance goals. One could argue that athlete’s academic performance is a joint responsibility of the academic units in the larger university and the athletic department. The admission of the athletes into various courses, and how the athletes are taught and evaluated in those courses are out of the purview of the athletic department. On the other hand, the athletic endeavor and performance (i.e., winning) is, to a great extent, dependent on the efforts and decisions made by athletic department. For example, the recruitment of student-athletes, the number of athletic scholarships, the number of coaches and their salaries, the budget allotted to different teams is all under the control of the athletic department. Therefore, it should not be surprising that the resources had greater impact on athletic performance than on academic performance.

It was surprising, however, that the level of resources was weakly related to gender equity. It must be noted that the goal of achieving gender equity is entirely in the hands of the athletic department while athletic and academic performances are largely in the hands of the athletes themselves. Further, gender equity is a relatively more stable attribute than the other two performance outcomes that fluctuate from year to year. One explanation for our results could be that due to the strict enforcement of Title IX provisions, most, if not all, athletic departments would have achieved or approaching the necessary gender equity. Any additional resources at hand would not affect gender equity, which is a finite attribute (i.e., it could not go beyond a 50–50 split) in the context of intercollegiate athletics in the U.S.

The distinction between intangible and tangible resources underscores the fact that generation of tangible resources is a short-term affair while intangible resources accrue over time. In addition, generation of measurable tangible resources which require concerted efforts from year to year is also dependent on the athletic success of the teams from year to year. In contrast, cultivating athletic and academic reputation is a long-term phenomenon and the success of the efforts in this regard cannot easily be judged. Despite these differences, both forms of resources and the outcomes are highly interrelated in a cyclical manner. As noted, intangible resources accrue to an athletic department over the long run as a function of the continued and consistent success from year to year in the generation of tangible resources which is largely dependent on how successful the athletic teams are in successive years in both the athletic and academic arenas. Stated otherwise, continued non-attainment of the desired outcomes (both athletic and academic) over the years may tarnish the reputation (i.e., the intangible resources) of the athletic department. But even more perilous is the fact that the attainment of the athletic goals through improper means may drastically reduce the tangible and intangible resources. Given that it takes a long time to build valuable intangible resources in the context of intercollegiate athletics, hence the administrators of athletic department have to be vigilant in promoting the long-term intangible reputation (both academic and athletic) of the university athletics while maintaining athletic integrity.

As Carmeli [34] noted, the tangible resources are more ‘flexible’ as opposed to more intangible resources which are ‘inflexible.’ Since flexible resources can be easily put to different uses (e.g., using the available money to buy equipment or pay for travel), organizations and their administrators are more likely to focus on tangible resources and ignore the intangible resources. In addition, as managers are often expected to be successful in the short run and are evaluated based on the annual successes, it might be inevitable that athletic directors may focus on tangible resources without considering the repercussions for the intangible resources over time. In this regard, it is useful to bear in mind Hall’s [36] distinction between fame (a short-term factor) and esteem (a long-term factor). That is, managers of athletic departments while seeking fame must also be cognizant of building the esteem over a period of time. While winning competitions and championships may engender fame, continued successes over the years and the legal and moral practices are the ingredients for long-term cultivation of esteem.

One of the differences between RBV and the traditional economics theory is that the RBV includes critical resources that do not comply with mathematical formulas of economics. Simply put, the RBV emphasizes the importance of unobservable factors that are not shown in the balance sheets of organizations [37, 54]. Especially, the theory of RBV argues that inimitable and non-substitutable resources are highly likely to be sources of competitive advantages because such resources can be barriers for other competitors [17, 54]. Since the theory itself comes with the importance of unobservable, or difficult to observe resources, it leads inevitably to the necessity of identifying and measuring the critical but intangible resources.

While most of the indicators of the variables in the model were direct measures, one of the indicators of institutional reputation was a proxy measure to refer to “tradition” or “history” of an athletic department. While our use of “years since establishment” may be considered a good proxy for tradition and history, future studies need to explore if there are other direct measures or even better proxy measures.

Because we were interested in testing the hypothesized structural model, we included all Division I schools (Division I-FBS, I-FCS, and non-football D-I institutions) in the analyses to meet the minimum number of observations required for SEM procedures. This overlooks fundamental differences among the three levels of schools within Division I. For instance, schools with a football program (as is the case with Division I-A or FBS schools) are likely to differ considerably in the magnitude of resources they have, and the dynamics of competitive advantage thereof. Future studies may use different statistical approaches which can test subgroup differences in regard to gaining competitive advantages. Also, this study used only a single year data. Future studies should also consider analyzing multi-year composite data or conducting time series analyses.

Future studies may also test the model of resources in other sport settings such as the professional sport leagues. However, it must be noted that professional sport franchises are concerned with only one sport whereas intercollegiate athletics deal with several sports. Further, intercollegiate athletics is also concerned with the dual and at times contradictory goals of performance and development of athletes.

While the present study has shown the influence of the various resources on attainment of the dual goals of athletic department goals, it did not address the issue of how well the available resources were utilized. It is one thing to say that a university has such and such resources and it is another thing to say how the managers of a university put those resources to different uses, and exploited the advantages accruing from those resources. The ability to exploit the available resources and reap the potential competitive advantage has been labeled “organizational capability” [16]. Future studies may include a measure of such capability and assess its contribution to competitive advantage.
